# Polysulfide Serves as a Hallmark of Desmoplastic Reaction to Differentially Diagnose Ductal Carcinoma In Situ and Invasive Breast Cancer by SERS Imaging

**DOI:** 10.3390/antiox12020240

**Published:** 2023-01-20

**Authors:** Akiko Kubo, Yohei Masugi, Takeshi Hase, Kengo Nagashima, Yuko Kawai, Minako Takizawa, Takako Hishiki, Megumi Shiota, Masatoshi Wakui, Yuko Kitagawa, Yasuaki Kabe, Michiie Sakamoto, Ayako Yachie, Tetsu Hayashida, Makoto Suematsu

**Affiliations:** 1 Departments of Biochemistry, Keio University School of Medicine, Tokyo 160-8582, Japan; 2 Department of Pathology, Keio University School of Medicine, Tokyo 160-8582, Japan; 3 The Systems Biology Institute, Tokyo 141-0022, Japan; 4 Biostatistics Unit, Clinical and Translational Research Center, Keio University Hospital, Tokyo 160-8582, Japan; 5 Department of Surgery, Keio University School of Medicine, Tokyo 160-8582, Japan; 6 Analysis Technology Center, CTO Office, FUJIFILM Corporation, Minamiashigara-shi 250-0193, Kanagawa, Japan; 7 Department of Laboratory Medicine, Keio University School of Medicine, Tokyo 160-8582, Japan; 8 Live Imaging Center, Central Institute for Experimental Animals, Kawasaki-shi 210-0821, Kanagawa, Japan

**Keywords:** breast cancer, imaging metabolomics, polysulfide, hypotaurine, glutathione

## Abstract

Pathological examination of formalin-fixed paraffin-embedded (FFPE) needle-biopsied samples by certified pathologists represents the gold standard for differential diagnosis between ductal carcinoma in situ (DCIS) and invasive breast cancers (IBC), while information of marker metabolites in the samples is lost in the samples. Infrared laser-scanning large-area surface-enhanced Raman spectroscopy (SERS) equipped with gold-nanoparticle-based SERS substrate enables us to visualize metabolites in fresh-frozen needle-biopsied samples with spatial matching between SERS and HE staining images with pathological annotations. DCIS (*n* = 14) and IBC (*n* = 32) samples generated many different SERS peaks in finger-print regions of SERS spectra among pathologically annotated lesions including cancer cell nests and the surrounding stroma. The results showed that SERS peaks in IBC stroma exhibit significantly increased polysulfide that coincides with decreased hypotaurine as compared with DCIS, suggesting that alterations of these redox metabolites account for fingerprints of desmoplastic reactions to distinguish IBC from DCIS. Furthermore, the application of supervised machine learning to the stroma-specific multiple SERS signals enables us to support automated differential diagnosis with high accuracy. The results suggest that SERS-derived biochemical fingerprints derived from redox metabolites account for a hallmark of desmoplastic reaction of IBC that is absent in DCIS, and thus, they serve as a useful method for precision diagnosis in breast cancer.

## 1. Introduction

Ductal carcinoma in situ (DCIS) was rarely diagnosed before the advent of breast screening, yet it now accounts for 25% of detected breast cancers [1,2]. DCIS is characterized by a proliferation of neoplastic luminal cells confined to the ducto-lobular system of the breast, which progresses towards invasive breast cancer (IBC). DCIS cells penetrate the ductal basement membrane to invade the surrounding stromal regions and result in IBC [3,4]. The invasiveness of breast cancer is a determinant of therapeutic strategies including mastectomy and/or systemic chemotherapy. It is thus critical to carry out precise S2 differential diagnosis between DCIS and IBC for decision making of therapeutics for patients. For diagnostic imaging, mammography and needle biopsy, which are coupled with pathological examination, constitute major approaches to diagnosis [5,6].

After DCIS detection, pathologists classify lesions by histologic features, including level of aggressiveness. Some studies showed a slight tendency for a high grade to progress towards IBC [2], while others demonstrated that grade is not significantly associated with the risk of the local invasive recurrence [7]. A quality of differential diagnosis to distinguish IBC from DCIS influences patients’ burden of therapeutics such as chemotherapy and surgical treatment. However, it highly depends on subjective judgement of morphological features of histological staining in the needle-biopsied samples by pathologists. Formalin-fixed paraffin-embedded (FFPE) needle-biopsied samples coupled with hematoxylin-eosin (HE) staining allows pathologists to achieve clear morphological information at cellular levels. On the other hand, such a pathological diagnosis may hamper objective judgement through marker metabolites to distinguish DCIS from IBC, since all small molecular metabolites are missing during the FFPE processes. Pathological diagnosis depends on manual judgement of individual cells including cancer and stromal cells by professional pathologists. Up to now, however, no automated subsidiary method supported by machine-readable systems has been established. The development of such methods helps pathologists achieve further accuracy of pathological diagnosis that resultantly benefits patients.

To overcome difficulties of differential diagnosis between DCIS and IBC, this study aimed to design objective and automated analyses of metabolites in needle-biopsied tissue samples derived from patients. To this end, we have applied large-area surface-enhanced Raman spectroscopy coupled with a two-dimensional gold nanoparticle array as a substrate, which enabled us to visualize multiple metabolites residing in frozen tissue samples [8,9,10]. The results suggest that SERS peaks detected in the stroma of IBC exhibit significantly increased signals of polysulfide, which coincides with decreased signals of hypotaurine as compared to DCIS, suggesting that the alterations of these redox metabolites in stroma account for fingerprints of desmoplastic reactions to distinguish IBC from DCIS. Furthermore, the application of machine-readable SERS data to extreme gradient boost (XGBoost)-based machine learning enables us to support accurate and automated differential diagnosis between IBC and DCIS.

## 2. Materials and Methods

### 2.1. Patients and Tissue Samples

This study was performed with the approval from the Internal Review Boards (IRBs) on ethical issues of Keio University School of Medicine and Hospital under written form of informed consents (ID: 20100143; PI: Associate Professor Masatoshi Wakui) and FUJIFILM Corporation (Kanagawa, Japan). The needle-biopsied samples were derived from patients who were suspected with breast cancer. One among multiple biopsied samples was immediately frozen with liquid nitrogen without FFPE preparation to prepare multiple 10 µm tissue slices that were used for SERS imaging according to our previous methods [8,9,10]. Other needle-biopsied specimens were prepared for FFPE samples to determine routine pathological diagnosis. After collecting SERS imaging data from a frozen sample, we used the same tissue slice for hematoxylin–eosin staining for pathological examination that secures a quality of diagnosis. The histological type of tumor was classified according to the World Health Organization criteria, based on immunohistochemical expressions of estrogen receptor (ER), progesterone receptor (PR), Ki-67 and HER2 with confirmation by in situ hybridization where appropriate. Histological grading was performed with reference to the grading systems proposed by previous studies [11]. Board-certified pathologists who have national license then confirmed the presence of cancer cells in all tissue samples. When it was difficult to distinguish DCIS from IBC, we separately prepared a semi-serial tissue section from FFPE-treated biopsied sample to stain p63, a molecule expressed in the outer layers of breast ducts (Figure S1); as seen, p63 in the individual breast ducts was maintained in DCIS, while being disappeared in IBC [12,13]. 

### 2.2. Au Nanoparticle-Based SERS Imaging with Infra-Red Laser Scanning Microcopy 

The fabrication of SERS substrates followed our previous studies [8,9,10]. As reported in the previous studies [9,10], the substrate was named gold (Au) nanofève (GNF) after the shape of Au nanoparticles that generate many electromagnetic hotspots as SERS excitation sources on the two-dimensional plate to enable large-area visualization of molecular vibration fingerprints of metabolites in tissue slices with sufficient sensitivity and uniformity [9,10]. The optical transparency of the GNF substrate allowed us to visualize the Raman signals by using an inverted-type laser confocal microscope. The inverted-type Raman microscopic system used in this study (Ramanforce; Nanophoton Co., Suita, Osaka, Japan). Use of 50× (NA = 0.65) objective lens provided optimal conditions such that the SERS signals could be maximally enhanced. The polychromatic device in the system enabled us to achieve SERS spectra under different conditions of diffraction grating with 300 grooves mm^−1^. The time for laser scanning was minimized by routinely acquiring SERS images at 300 grooves mm^−1^, unless otherwise mentioned. Under the routine conditions of the 50× objective lens, a horizontally lining laser beam at a width of 186 µm (=400 pixels/line) was scanned vertically, and the signals yielded from 100 pixels were binned to output the signals per 4 pixels line^−1^ [10]. For high resolution image acquisitions, 20 pixels were binned to output the signals per 20 pixels line^−1^. Finally, a series of the laser scanning stripe images was tiled to cover the entire microscopic fields which pathologists examined. 

All spectral data were stored at individual 100-binned pixels into the computer storage. Under these circumstances, the system allowed spatial resolution of 46.6 µm pixel^−1^, unless otherwise mentioned. As described previously [9,10], the sensitivity and frequency of the microscope system were calibrated using the Raman shift yielded by silicon phonon mode at 520 cm^−1^. Prior to SERS measurements, Raman shift in silicon phonon mode were determined for calibration. The background noise of SERS signals was subtracted from the recorded spectra by using the weighted mean fitting with Lanczos second function [9,10]. The resolutions of wave numbers were about 2 cm^−1^, when the grating of 300 grooves mm^−1^ was used for SERS measurements, respectively. Metabolites in the air-dried tissue sections were visualized by mounting 5 µm-thick tissue sections on GNF substrates and placing them in the vacuum dry chamber. To achieve a quality of SERS images with greater signal–noise ratios, we accumulated SERS signals at the central peak wave number of ±5 cm^−1^.

We further investigated the spatial relationship between SERS imaging and HE staining, which were acquired after SERS imaging and were annotated to identify cancer cell nests and the surrounding stromal regions by a board-certified pathologist. As mentioned previously, immediately after the SERS images of tissue sections were recorded, the same sections were stained with HE staining. The microscopic images of HE-stained tissue sections mounted on optically transparent GNF substrates were directly imported as digital photo files by using NanoZoomer v.2.0-HT (Hamamatsu Photonics, Hamamatsu, Japan). Cancer cells were annotated by manually tracing cancer cell nests by certified professional pathologists at the magnification of 10× or 20× by using a 27-inch monitor and NDP view2 software (Hamamatsu Photonics, Hamamatsu City, Shizuoka, Japan), while carefully excluding non-cancer cells and structures, including any extracellular materials. Invasive cancer nests were generally distinguished from non-invasive cancer nests using HE-stained slides based on microscopic findings, including the presence of an irregular growth pattern, a haphazard architecture, and a desmoplastic stroma. An additional immunohistochemical analysis of a myoepithelial marker p63 was performed to determine whether given lesions were invasive, when the diagnosis was problematic in HE-stained slides. We also annotated non-cancerous stroma within the cancer tissues by using the identical method described above to trace non-cancerous stromal regions instead of tracing cancer cell nests. The non-cancerous stroma is defined as stromal regions that do not contain any cancer cells within cancer tissues. If candidate regions of the stroma in cancer tissues include cancer cells that disturb the annotation of the stroma, such regions were excluded from the annotation. Cancer tissues are defined as an area surrounded by the borderline of the invasive margin of cancer cells. 

### 2.3. SERS Data Processing for Machine Learning to Differentially Diagnose DCIS and IBC

All spectral data stored at individual 100-binned pixels were processed by using IMAGEREVEAL MS software (Shimadzu Co., Kyoto, Japan). The microscopic images of HE-stained tissue sections with annotation were overlayed on SERS images, then spectral data located in the annotated areas were tagged. The tagged spectral data were output as text files (x, y, annotation tag, spectrum). To perform statistical analysis, tagged spectra were averaged per patient, and Student’s *t*-test was performed. Among the significantly different wave numbers, 6 peak wave numbers of ±10 cm^−1^ were compared with clinical tag information. Continuous and categorical variables were compared using Welch’s *t*-test and Fisher’s exact test, respectively. Odds ratios (ORs) were estimated using univariable and multivariable logistic regression models [14]. Possible prognostic factors (i.e., age, and triple negative) were adjusted in multivariable analyses. A *p*-value of <0.05 was considered statistically significant. R version 4.2.1 (R Foundation for Statistical Computing, Vienna, Austria) was used for all statistical analyses.

A total of 46 patients consisting of 32 invasive breast cancer patients and 14 DCIS patients (Table 1) were first randomly stratified and split into 4 sets of 10 patients each so that the class distribution was balanced. Among 4 sets of the data, 3 sets were used for model training, and 1 set was kept for model evaluation. SERS imaging data containing stromal regions in cancer tissues were digitally processed to be covered with a mesh (46.6 µm pixel^−1^) which is designated as regions of interests (ROIs). ROIs in each patient in the training sets were collected with the corresponding class labels. As shown later in Results, the intensity values of 27 differentially expressed SERS signals between invasive and DCIS patients in each mesh were utilized as features of the following supervised classification. Multiple machine learning algorithms were applied and screened including L1 regularized logistic regression (L1) [15], support vector machines (SVM) [16], random forest (RF) [17], extreme gradient boosting (XGBoost) [18,19,20], k-nearest neighbors (KNN) [21] and Naïve Bayes (NB) [22]. The classifier of each algorithm was trained by three-fold cross-validation, and the hyper parameters were optimized with grid search to minimize kappa. The best-performing model in each algorithm was refitted with the test fold in the cross-validation. The best-performing model among 6 algorithms was determined with the evaluation set containing 10 patients whose data were not utilized in model training. Collectively, a schematic diagram showing SERS imaging, pathological annotation and machine learning system is shown in Figure S2.

### 2.4. Imaging Mass Spectrometry

A MALDI-TOF mass spectrometer (iMscope, Shimadzu Co., Kyoto, Japan) was used for 25 μm interval imaging mass spectrometry [23]. The mass peaks of metabolites collected from tissue sections were identified by comparing MS/MS fragment patterns with those collected from standard reagents. A matrix compound used for ionization was 9-aminoacridine (Merck, Darmstadt, Germany), according to our previous method [24,25,26].

### 2.5. Statistical Analyses

Continuous and categorical variables were compared using Welch’s *t*-test and Fisher’s exact test, respectively. Odds ratios (ORs) were estimated using univariable and multivariable logistic regression models [14]. Possible prognostic factors (i.e., age, and triple negative) were adjusted in multivariable analyses. Cutoff values were established based on three-fold cross-validation. A *p*-value of <0.05 was considered statistically significant. R version 4.2.1 (R Foundation for Statistical Computing, Vienna, Austria) was used for all statistical analyses.

## 3. Results

### 3.1. Comparative Characterization of SERS Spectra in DCIS and IBC

Table 1 indicates the list of patients with breast cancer (*n* = 46) which include 14 DCIS and 32 invasive breast cancer (IBC). 

**Figure 1:**
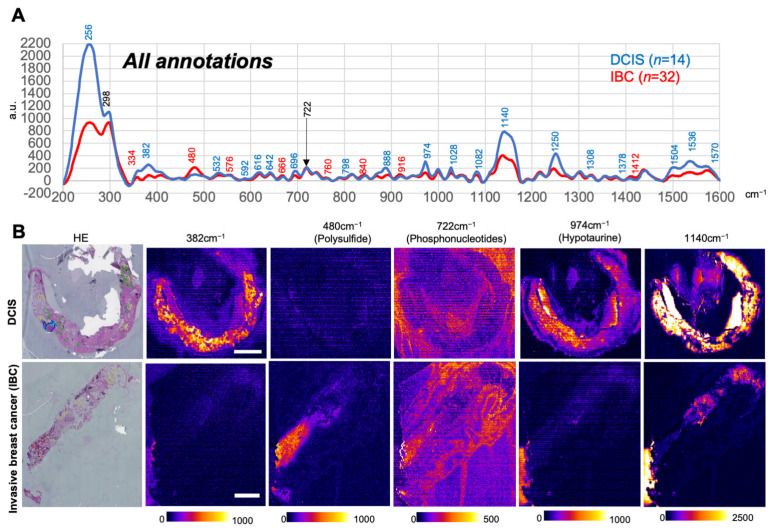
On-tissue visualization of Raman scattering signals by SERS imaging in tissue sections derived from ductal carcinoma in situ and invasive breast cancer. (**A**) Averaged SERS spectrum from ductal carcinoma in situ (DCIS; *n* = 14) and invasive breast cancer (IBC; *n* = 32). Spectra in blue and red indicate those of DCIS and IBC, respectively. Spectra in blue and red indicate those of DCIS and IBC. Significant differences between DCIS and IBC are marked in blue and red depicted greater SERS signals in DCIS and IBC, respectively, while the wave number indicated in black showed no significant differences between the two groups. SERS signal intensities are expressed as arbitrary units (a.u.) with pseudo-color representation. Data were accumulated from all annotated regions including cancer cell nests and stroma. (**B**) Representative pictures of HE staining and SERS imaging at 382 ± 5 cm^−1^, 480 ± 5 cm^−1^, 722 ± 5 cm^−1^, 974 ± 5 cm^−1,^ and 1140 ± 5 cm^−1^ in DCIS and invasive breast cancer at spatial resolutions of 46.7 µm per pixel. After SERS image acquisition, HE staining (HE) was performed in the same tissue slice. Annotations in HE staining show cancer cells in red, cancer stroma in white, DCIS in light blue, DCIS stroma in dark blue, fiber in green, fat in yellow, and normal mammalian grand in black lines. Bars indicate 1 mm.

Between DCIS and IBC groups, no significant differences were seen in median year of diagnosis, age of diagnosis and clinic-pathological features. Figure 1A illustrates the mean SERS spectra of DCIS and IBC, which include all annotated regions, and were shown in blue and red, respectively. The wave numbers which showed statistical differences were listed as 19 D-dominant (DCIS > IBC) and 8 I-dominant (IBC-dominant, DCIS < IBC) peaks in Table S1.

Among these SERS peaks, representative SERS images visualized with low magnification are shown in Figure 1B. Results indicate that polysulfide identified at 480 cm^−1^ is significantly greater in IBC than in DCIS, while hypotaurine at 974 cm^−1^ is significantly greater in DCIS than in IBC. The peak at 722 cm^−1^ did not differ between DCIS and IBC. Since this peak indicated the presence of high-energy purine phosphor-nucleotides such as ATP and ADP, the results suggest that viability of the frozen tissue samples was maintained [8]. Figure S3 illustrates dot plots of individual data at each wave numbers, indicating that 27 specific wave numbers were identified in fingerprint regions of SERS spectra to distinguish DCIS from IBC. As described later in Results, the peak at 382 cm^−1^ was unassignable but accounted for the most reliable hallmark of differential diagnosis of DCIS which disappeared in IBC.

**Figure 2:**
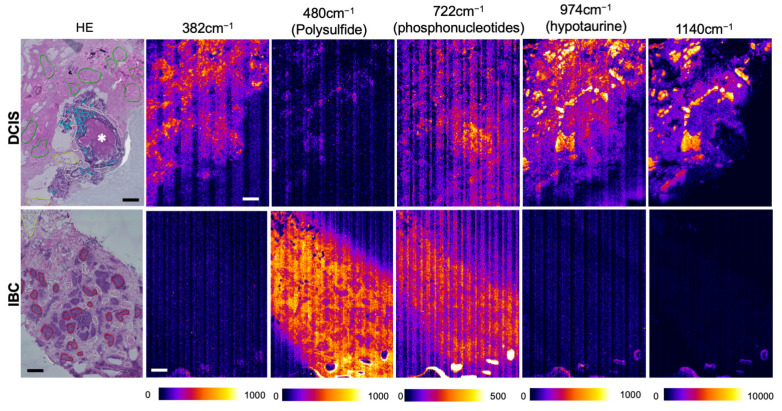
Representative SERS images indicating increased polysulfide signal at 480 cm^−1^ in IBC under high spatial resolution. SERS images were captured at 382 ± 5 cm^−1^, 480 ± 5 cm^−1^ (polysulfide), 722 ± 5 cm^−1^ (purine phospho-nucleotides), 974 ± 5 cm^−1^ (hypotaurine), and 1140 ± 5 cm^−1^ in DCIS and IBC at spatial resolutions of 9.34µm per pixel. Individual line-scanning SERS images were automatically tiled as shown in the panels. Pseudo-color representations indicate increasing SERS intensities which are expressed as arbitrary units (a.u.). HE staining was performed after SERS image acquisition in the same tissue slice. Annotations in HE staining show cancer cells in red, cancer stroma in white, DCIS in light blue, fiber in green, adipose tissues in yellow, and normal mammalian grands in black lines. Bars indicate 200 µm. Vertical stripes indicate optical aberration caused by laser scanning and tiling images under high-power magnification. * Calcification in DCIS.

To further clarify the relationship between SERS signals and pathological annotations judged by HE staining, the SERS images were captured at higher spatial resolution. As seen in Figure 2, several images captured at specific wave numbers displayed contrast differences between DCIS and IBC. Among them, the polysulfide signal at 480 cm^−1^ exhibited greater intensities in IBC than in DCIS. By contrast, the signal in IBC displayed a modest increase in the signal in cancer cell nests that coincided with a remarkable elevation in the surrounding stromal regions. As a result, the elevation of polysulfide signal cancer-associated stromal regions accounted for an important feature of desmoplastic reactions that occur in the stroma of IBC. On the other hand, the hypotaurine signal at 974 cm^−1^ and the unidentified signal at 1140 cm^−1^ colocalized each other, and the spot signals appeared to occur along the DCIS cells, which were featured by marking in light blue (Figure 2). The site of calcification did not exhibit the hypotaurine signal (see asterisk). Note that these results suggest that the pattern of reactive sulfur species differs between DCIS and IBC.

### 3.2. Stroma-Specific SERS Analyses Distinguish IBC from DCIS

**Figure 3:**
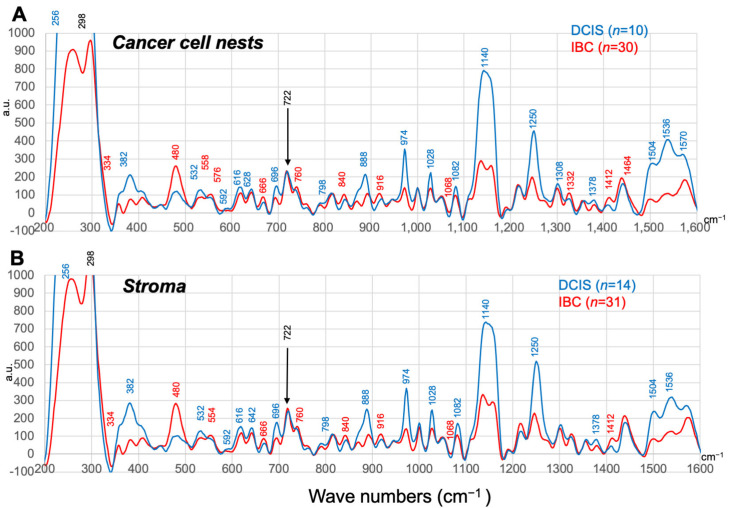
(**A**) The averaged SERS spectra from cancer cell nest regions of DCIS (blue, *n* = 10) and those of invasive breast cancer (IBC: red *n* = 30). (**B**) The averaged SERS spectra from stroma regions of DCIS (blue, *n* = 14) and those of invasive breast cancer (IBC: red *n* = 31). Intensities are expressed as arbitrary units (a.u.). Peaks showing significant differences between DCIS and invasive cancer are marked in blue (DCIS > IBC) and red (DCIS < IBC), respectively.

Observations suggesting desmoplastic reactions in IBC led us to further examine whether SERS spectral profiles may differ among different pathological annotations. Figure 3 illustrates mean SERS spectra collected from cancer cell nests and stromal regions, respectively. Dot-plotting graphs and tables indicating statistical parameters to show differences between DCIS and IBC in cancer cell nests and stroma were shown separately in dot-plotting presentation in Figures S4 and S5, and in statistical comparison in Tables S2 and S3, respectively. We also inquired whether a clinically used classification of breast cancer (Luminal A and B, Her2, triple negative) may influence the SERS peaks. Based on the data shown in Tables S4–S6, univariable logistic regression analyses were carried out. As seen in Table S7, there was no significant correlation between the classification of breast cancer and the choice of annotations. 

Based on these data, we chose five important wave numbers (382 cm^−1^, 480 cm^−1^, 974 cm^−1^, 1140 cm^−1^, 1250 cm^−1^) in fingerprint regions that are useful to distinguish DCIS from IBC, while the peak at 722 cm^−1^ served as a control indicating purine phosphonucleotides to examine through univariable and multivariable analyses (Table 2 and Table 3). As seen, the analyses revealed that the SERS signals at the five specific wave numbers measured in stromal regions were all significant to discriminate IBC from DCIS, while those measured in cancer cell nests at 480 cm^−1^ were not significant. These results led us to examine whether the SERS data extraction from stromal regions, but not from cancer cell nest regions, may benefit differential diagnosis between DCIS and IBC through machine learning. 

### 3.3. SERS Spectra of Adipose Tissues in IBC Differ from Those in DCIS

Besides cancer cell nests and stromal regions, we examined whether adipose tissues may differ between DCIS and IBC. Figure S6 illustrates differences in mean SERS spectra between DCIS (in blue) and IBC (in red), which were coupled with statistical significance of the differences in Table S8, respectively. Careful observation revealed the absence of difference in the SERS peak at 480 cm^−1^, suggesting that polysulfide occurring in the adipose tissues does not differ between DCIS and IBC. While many SERS peaks were detected in DCIS, those peaks disappeared in IBC to result in many DCIS-dominant peaks (D-dominant peaks in Table S8). These results suggest that small molecular metabolites which are detected as the fingerprint regions of SERS spectra in DCIS are significantly decreased in IBC.

### 3.4. XGB Model Enables Us to Provide Reliable Machine Learning to Translate SERS Data

As mentioned previously in Table 2 and Table 3, SERS data extraction from stromal regions may represent differences in metabolic changes between DCIS and IBC, thus benefiting their differential diagnosis. Among six algorithms shown in Materials and Methods [15,16,17,18,19,20,21,22], the best-performing model was determined with the evaluation set of data collected from 10 patients whose data were not utilized in the model training. Table 4 illustrated a comparison of predictive performance among six representative classification algorithms in cross-validation of SERS signals collected from stromal regions. 

As judged by Cohen’s kappa scores and accuracy values, the XGB model displayed the most excellent profiles of machine learning. Table S9 showed the accuracy of the prediction of the XGB model on the test-fold dataset in three-fold cross-validation. The confusion matrix in this table indicated that errors occurred only in 7 meshes versus 2819 meshes in stromal regions of IBCs, while 13 meshes versus 298 meshes were incorrect in DCIS. 

Under these conditions, data collected from 10 patients (7 IBC cases and 3 DCIS cases) whose data were not used in training were tested by XGB model (Table 5).

As seen, all seven IBC and three DCIS patients were correctly classified where the mesh-level prediction accuracy was as high as more than 95% in all patients. The XGB model allowed us to know which wave number of the SERS spectrum in stromal regions exhibits the feature importance in distinguishing DCIS from IBC in machine learning. As seen in Table 6, the SERS signals at 382 cm^−1^ (unidentified), 974 cm^−1^ (hypotaurine) and 480 cm^−1^ (polysulfide) were the three highest-ranking wave numbers in terms of importance in determining the prediction. These results suggest that machine-readable SERS imaging data in stromal regions of breast cancer benefit automated differential diagnosis between DCIS and IBC.

### 3.5. Detection of Polysulfide/Persulfide in Breast Cancer Tissues by Imaging Mass Spectrometry (IMS)

**Figure 4:**
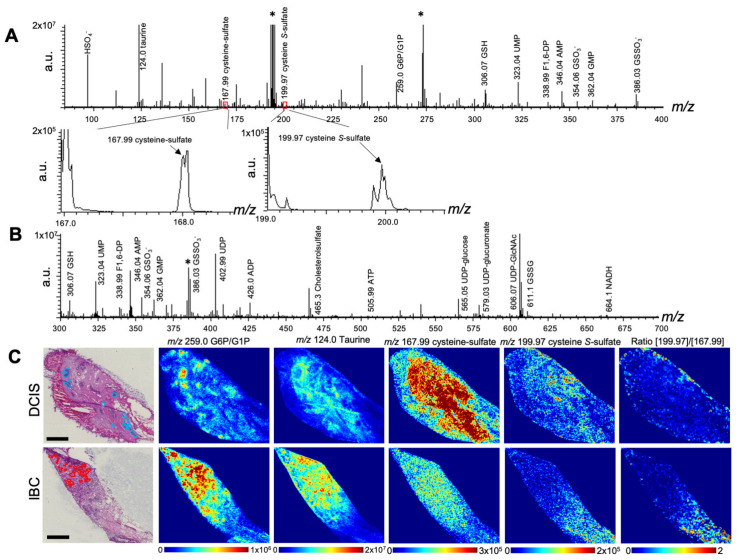
Imaging mass spectrometry of tissue sections derived from ductal carcinoma in situ (DCIS) and invasive breast cancer (IBC). (**A**) Averaged mass spectrum of needle biopsy specimen in negative mode in a range of *m*/*z* 85–400. Left and right insets indicate magnified spectrum around *m*/*z* 168.0 (cysteine sulfate) and that around *m*/*z* 200 (cysteine *S*-sulfate) (**B**) Averaged mass spectrum in a range of *m*/*z* 300–700, respectively. * Artifacts yielded by matrix. (**C**) Representative pictures of HE staining and MALDI-mass spectrometric imaging at *m*/*z* 259.0, *m*/*z* 124.0, *m*/*z* 167.99, *m*/*z* 199.97, and ratio image of [199.97]/[167.99] in DCIS and IBC at spatial resolutions of 25 µm per pixel. After capturing mass spectrometric images, HE staining was performed in the same tissue slices. Annotations in HE staining show cancer cells in DCIS in blue and those in IBC in red. Bars indicate 1 mm.

SERS imaging contributes to non-invasive chemical diagnosis on the tissue. However, this strategy is based on the chemical fingerprint/profile other than a set of separate metabolite markers, and each wavenumber may be contributed by multiple species carrying the same functional groups. The peak at 480 cm^−1^ accounts for metabolites carrying sulfane–sulfur bond(s), since the signal disappears in response to monobromobimane, an electrophile compound breaking the sulfane–sulfur bond (10). We were thus challenged to detect cysteine persulfide, one of polysulfide species that generates the SERS peak at 480 cm^−1^ (10). As seen in Figure 4, IMS allowed us to detect accumulation of G6P/G1P in cancer cell nests of DCIS (light blue) and IBC (red) Furthermore, the signal was likely to be larger in IBC than in DCIS, presumably because of activation of the Warburg effect that not only drives lactate formation but also accelerates serine synthesis and trans-sulfuration pathway. IMS causes irreversible auto-oxidation of redox metabolites, such as hypotaurine into taurine, cysteine into cysteine-sulfate, and cysteine persulfide into cysteine *S*-sulfate. Figure 4A indicated the peak of cysteine *S*-sulfate, suggesting the presence of cysteine persulfide in both DCIS and IBC. The ratio imaging of cysteine *S*-sulfate versus cysteine-sulfate, which suggests enrichment of persulfide species [26], showed predominant distribution in cancer-associated stromal regions of IBC. These results were in good agreement with SERS imaging data (Figure 2).

## 4. Discussion

On-tissue visualization of metabolites using Au nanoparticle-based SERS imaging is an imaging metabolomics method that does not require labelling or staining of tissue slices. The SERS substrate which we developed is composed of two-dimensional arrays of many Au nanoparticles in the surface area of 24 mm × 24 mm and is thus suitable for pathological diagnosis using frozen tissue sections. Au nanoparticle-based SERS substrate enables us to capture many redox metabolites including glutathione, hydrogen persulfide, polysulfide and hypotaurine in cancer tissues [9,10]. The current study demonstrated that tissue slices derived from frozen needle-biopsied samples allowed us to detect these sulfur-containing redox metabolites. Furthermore, careful profiling of the SERS spectra provided key fingerprints of wave numbers for differential diagnosis of DCIS and IBC. At the same time, a significant increase in polysulfide that coincides with a decrease in hypotaurine in stromal regions of breast cancer plays a key event in IBC, and thus, it shows the presence of desmoplastic reactions under increased invasiveness. Pathologists carefully check the presence of desmoplastic reactions by microscopic examination through visually confirmation of unusual staining of eosin and/or irregular patterns of fibers in the stromal regions. To our knowledge, for the first time, the present results provided objective and biochemical evidence of desmoplastic reactions occurring in the cancer-associated stromal regions.

To further improve objectiveness and accuracy of diagnosis by SERS imaging, this study took on the challenge of accomplishing automated diagnosis by utilizing machine learning. Among six different classification algorithms (Table 4), the XGB model exhibited satisfactory accuracy with objectiveness and without misdiagnosis. To be noted is the observation that subtype classification of breast cancer is not associated with intensities of SERS peaks in specific annotations such as cancer cell nests or stroma (Table S7). The number of patients examined in this study is still low and requires more clinical cases to improve the quality of pathological diagnosis for clinical use. However, automated pathological diagnosis supported by SERS and machine learning should deserve further investigation if differences in long-term prognosis between patients with DCIS and those with IBC are monitored in clinical cases [27,28].

Our previous studies revealed the roles of polysulfide in cancer development; the same SERS imaging system enabled us to visualize polysulfide [9,10]. Endogenous generation of polysulfide plays a protective role in cancer cell survival, as ambroxol, a scavenger of polysulfide that decreases cell viability [10]. Furthermore, in ovarian clear cell carcinoma, cystathionine γ-lyase, an enzyme generating cysteine hydropersulfide, are highly expressed in cancer cells and determine overall survival of patients after the debulking surgery [10]. SERS imaging in frozen slices of the cancer exhibited polysulfide not only in cancer cells but also in stromal regions. Polysulfide generation appears to play a role in cancer chemoresistance through its inhibitory action on DNA-intercalation by anti-cancer reagents such as cisplatin [10]. On the other hand, hypotaurine is a potent antioxidant generated from cysteine. In cancer cells, the elevation of oxidative stress reduces hypotaurine to be converted to taurine, suggesting it is a negative marker of oxidative stress [9]. It is not unreasonable to suggest that cancer cells of IBC, as compared with those of DCIS, may upregulate reactive sulfur species (Figure 4), as compared with DCIS cells. Although the detailed mechanisms remain unknown, the invasiveness of breast cancer is highly associated with non-cancerous regions of cancer tissues which include not only stromal regions but also adipose tissues surrounding the cancer cells (Figure S5 and Table S9). Breast cancer develops in fatty acid-rich niche areas and actively regulates genes related to fatty acid metabolism in the microenvironments [29]. Further investigation is obviously necessary to unveil the genes and enzymes responsible for local regulation of polysulfide and hypotaurine, and to determine how the balance between these two redox metabolites determine breast cancer development.

## Figures and Tables

**Table 1:** Clinicopathological characteristics of patients used in this study (*n* = 46).

Characteristic	Type	Total	DCIS	IBC	*p*-Value
Number of patients		46	14	32	
Median year of diagnosis		2011	2010	2012	
		(2009–2012)	(2009–2012)	(2009–2012)	
Mean age of diagnosis		58.0	52.3	60.5	0.0781
		(34–87)	(34–78)	(34–87)	
Stage	0	14	14	0	
	I	18	0	18	
	IIA	11	0	11	
	IIB	2	0	2	
	IIIB	1	0	1	
Histology	DCIS	14	14	0	
	IDC	28	0	28	
	ILC	1	0	1	
	mucinous	1	0	1	
	type unknown	2	0	2	
Tumor size (T)	Tis	14	14	0	
	T1	21	0	21	
	T2	9	0	9	
	T3	1	0	1	
	T4	1	0	1	
Nodal status (pN)	N0	41	14	27	0.3034
	N1	5	0	5	
Metastasis status	M0	46	14	32	1
	M1	0	0	0	
Estrogen receptor status	ER+	34	11	23	0.7294
	ER^−^	12	3	9	
Progesterone receptor status	PR+	31	8	23	0.4952
	PR^−^	15	6	9	
HER2 status	HER2+	8	1	7	>0.9999
	HER2^−^	21	2	19	
	Not determined	17	11	6	
Type	luminal	36	11	25	>0.9999
	Non-luminal	10	3	7	
Nuclear grade	1	15	1	14	0.150
	2	6	2	4	
	3	5	0	5	
	Not determined	20	11	9	
Ki67	<14%	6	0	6	
	>14%	20	1	19	
	Not determined	20	13	7	
End-status	Alive	41	12	29	>0.9999
	Breast cancer death	0	0	0	
	Other death	1	0	1	
	Unknown	4	2	2	

DCIS (Non-invasive ductal carcinoma in situ), IDC (invasive ductal carcinoma), ILC (invasive lobular carcinoma), mucinous (mucinous carcinoma). Mean age; Unpaired *t*-test, Others; Chi square and Fisher’s exact test.

**Table 2:** Logistic regression models for invasive tumor (univariable analyses).

Characteristic		*n*	EVENT *n*	OR ^1^	95% CI ^2^	*p*-Value ^2^
382 +/− 10 cm^−1^	Whole annotated tissue region	46	32	0.99	0.98, 0.99	<0.001
	Cancer cell nest only	40	30	0.98	0.97, 0.99	<0.001
	Stroma region only	45	31	0.98	0.97, 0.99	<0.001
480 +/− 10 cm^−1^	Whole annotated tissue region	46	32	1.01	1.00, 1.03	0.015
	Cancer cell nest only	40	30	1.01	1.00, 1.02	N.S.
	Stroma region only	45	31	1.02	1.01, 1.03	0.010
974 +/− 10 cm^−1^	Whole annotated tissue region	46	32	0.98	0.97, 0.99	<0.001
	Cancer cell nest only	40	30	0.98	0.97, 0.99	0.003
	Stroma region only	45	31	0.99	0.98, 0.99	<0.001
1140 +/− 10 cm^−1^	Whole annotated tissue region	46	32	1.00	0.99, 1.00	0.013
	Cancer cell nest only	40	30	1.00	0.99, 1.00	0.005
	Stroma region only	45	31	1.00	0.99, 1.00	0.003
1250 +/− 10 cm^−1^	Whole annotated tissue region	46	32	0.99	0.99, 1.00	0.021
	Cancer cell nest only	40	30	1.00	0.99, 1.00	0.032
	Stroma region only	45	31	1.00	0.99, 1.00	0.022
722 +/− 10 cm^−1^	Whole annotated tissue region	46	32	1.00	0.99, 1.00	N.S.
	Cancer cell nest only	40	30	1.00	0.99, 1.01	N.S.
	Stroma region only	45	31	1.00	0.99, 1.01	N.S.

^1^ OR = Odds Ratio, ^2^ CI = Confidence Interval.

**Table 3:** Logistic regression models for invasive tumor (multivariable analyses).

Characteristic		*n*	EVENT *n*	OR ^1^	95% CI ^2^	*p*-Value ^2^
382 +/− 10 cm^−1^	Whole annotated tissue region	46	32	0.99	0.98, 0.99	<0.001
	Cancer cell nest only	40	30	0.98	0.97, 0.99	0.001
	Stroma region only	45	31	0.98	0.97, 0.99	<0.001
480 +/− 10 cm^−1^	Whole annotated tissue region	46	32	1.02	1.00, 1.03	0.018
	Cancer cell nest only	40	30	1.01	1.00, 1.02	N.S.
	Stroma region only	45	31	1.02	1.01, 1.03	0.010
974 +/− 10 cm^−1^	Whole annotated tissue region	46	32	0.98	0.97, 0.99	0.001
	Cancer cell nest only	40	30	0.99	0.97, 0.99	0.004
	Stroma region only	45	31	0.99	0.98, 0.99	0.002
1140 +/− 10 cm^−1^	Whole annotated tissue region	46	32	1.00	0.99, 1.00	0.030
	Cancer cell nest only	40	30	1.00	0.99, 1.00	0.006
	Stroma region only	45	31	1.00	0.99, 1.00	0.008
1250 +/− 10 cm^−1^	Whole annotated tissue region	46	32	0.99	0.99, 1.00	0.018
	Cancer cell nest only	40	30	1.00	0.99, 1.00	0.039
	Stroma region only	45	31	1.00	0.99, 1.00	0.021

^1^ OR = Odds Ratio, ^2^ CI = Confidence Interval.

**Table 4:** Predictive performance of the six representative classification algorithms in cross-validation.

Model Name	Kappa	Accuracy	F1	Precision	Recall
XGB	0.96	0.99	0.99	0.99	0.99
RF	0.92	0.99	0.99	0.99	0.99
SVC	0.91	0.98	0.98	0.99	0.98
NB	0.89	0.98	0.98	0.98	0.98
KNN	0.68	0.96	0.95	0.98	0.96
L1	0.88	0.98	0.98	0.98	0.98

Cohen’s kappa, accuracy, weighted F1, weighted precision, and weighted recall scores were used for evaluation.

**Table 5:** Predictive results of 10 patients with breast cancer (7 IBC and 3 DCIS cases) by XGBoost algorithm.

Case	Diagnosis	Prediction-IBC	Prediction-DCIS	Ratio of IBC	Predicted-Category
IBC-1	IBC	157	4	0.975	IBC
IBC-2	IBC	1398	3	0.998	IBC
IBC-3	IBC	579	0	1.000	IBC
IBC-4	IBC	185	5	0.974	IBC
IBC-5	IBC	228	0	1.000	IBC
IBC-6	IBC	54	0	1.000	IBC
IBC-7	IBC	2111	1	0.995	IBC
DCIS-1	DCIS	6	138	0.042	DCIS
DCIS-2	DCIS	1	49	0.020	DCIS
DCIS-3	DCIS	0	98	0.000	DCIS

The prediction of the XGBoost model on the stroma-ROI meshes of the evaluation patient set. All the seven IBC patients and three DCIS patients were correctly classified where the mesh-level prediction accuracy was as high as 95% in all patients.

**Table 6:** Evaluation of individual SERS peaks measured at stromal regions in XGBoost feature importance.

Wave Number (cm^−1^)	XGB Feature Importance
382	0.220
974	0.191
480	0.119
256	0.112
888	0.070
1250	0.040
334	0.038
1504	0.033
1028	0.024
840	0.022
916	0.022
1536	0.014
1412	0.014
696	0.013
760	0.009
1140	0.009
616	0.009
1378	0.008
642	0.006
592	0.006
1308	0.005
532	0.004
666	0.003
798	0.003
1570	0.003
576	0.002
1082	0.002

The feature importance of XGBoost model was calculated using permutation-based importance measure to investigate which SERS signals affect the most in classification of stroma.

## Data Availability

Data is contained within the article and supplementary material.

## Supplementary Materials

The following supporting information can be downloaded at: https://www.mdpi.com/article/10.3390/antiox12020240/s1, Figure S1: Immunostaining of p63 and HE staining of breast needle biopsy frozen sections; Figure S2: A schematic diagram of SERS imaging and Imaging MS equipped with data processing and machine learning systems; Figure S3: SERS signal intensities from whole annotated regions; Figure S4: SERS signal intensities from cancer cell nest; Figure S5: SERS signal intensities from stromal region; Figure S6: Averaged SERS spectra from adipose tissues; Table S1: Lists of Raman shifts occurring dominantly in frozen section of invasive tumors and those in DCIS in the human breast cancer tissue; Table S2: Lists of Raman shifts occurring dominantly in frozen section of invasive tumors and those in DCIS in the human breast cancer cell nest regions; Table S3: Lists of Raman shifts occurring dominantly in frozen section of invasive tumors and those in DCIS in the human breast cancer stroma regions; Table S4: Baseline data (whole annotated region) for univariable logistic regression analyses; Table S5: Baseline data (cancer cell nest) for univariable logistic regression analyses; Table S6: Baseline data (stromal regions) for univariable logistic regression analyses; Table S7: Logistic regression models for invasive breast cancer (univariable analyses); Table S8: Lists of Raman shifts occurring dominantly in frozen section of invasive tumors and those in DCIS in the human breast cancer adipose tissue; Table S9: RESULT in model training.
