# Supplementary material for: Polysulfide Serves as a Hallmark of Desmoplastic Reaction to Differentially Diagnose Ductal Carcinoma In Situ and Invasive Breast Cancer by SERS Imaging
Source: Antioxidants (Basel). 2023 Jan 20;12(2):240. doi: 10.3390/antiox12020240 (PMC9952617; doi:10.3390/antiox12020240)
Supplement: Supplementary file 1 [file antioxidants-12-00240-s001.zip › antioxidants-2169005-supplementary.pdf]

Figure S1

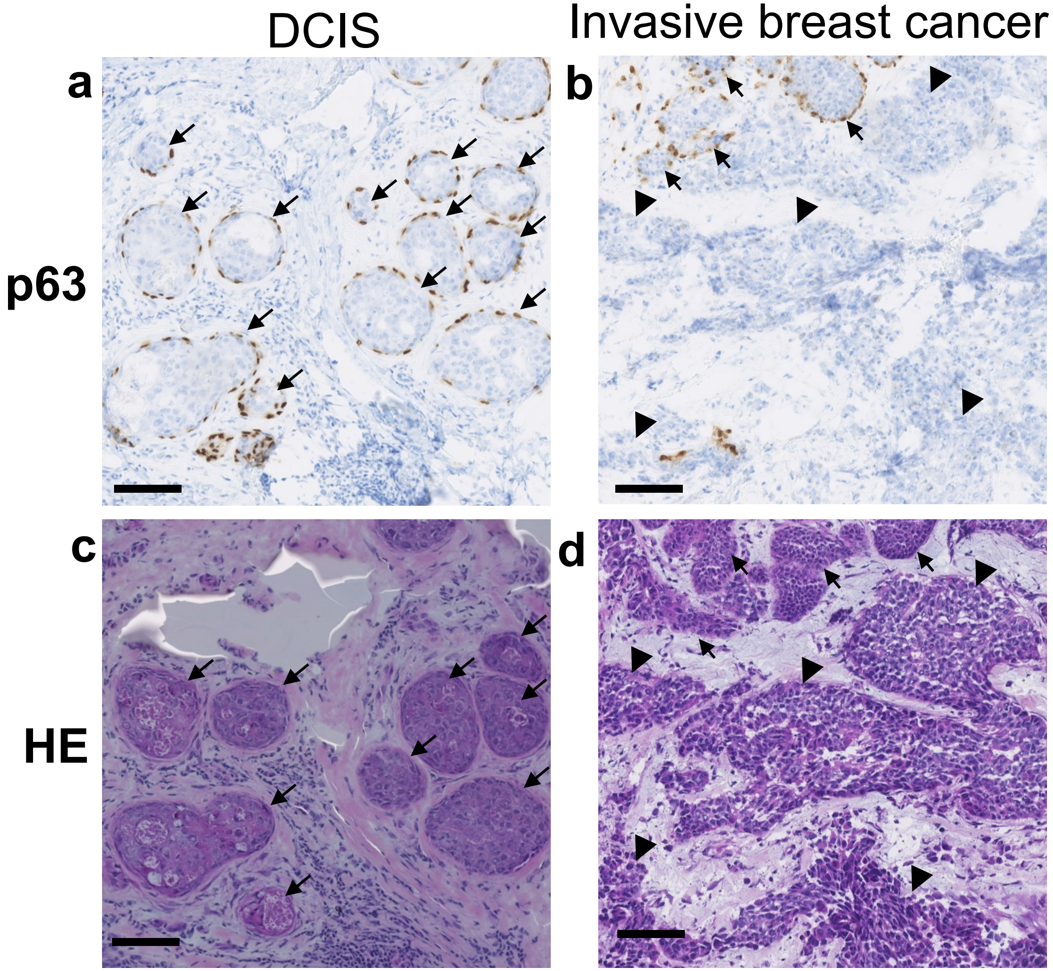

Immunostaining of p63(a,b) and HE(c,d) staining of breast needle biopsy-derived frozen sections. DCIS (a,c) and DCIS with interposed nests of invasive breast cancer (b,d). Arrows indicate nests of DCIS, Arrowheads indicate invasive cancer cells. Bar: 100μm

Figure S2

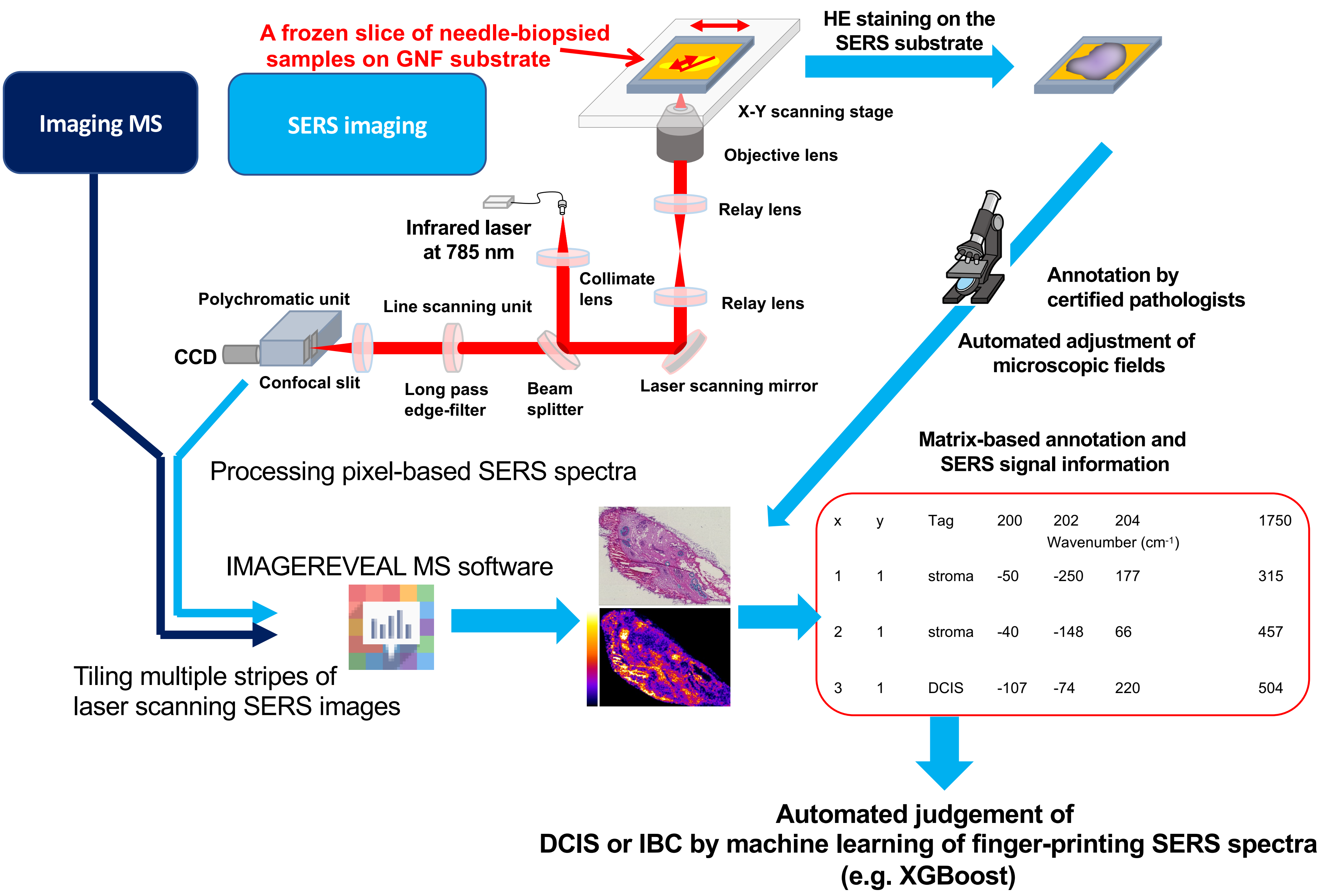
A frozen slice of needle-biopsied samples on GNF substrate
HE staining on the SERS substrate
X-Y scanning stage
Imaging MS
SERS imaging
Objective lens
Relay lens
Infrared laser at 785 nm
Collimate lens
Relay lens
Polychromatic unit
Line scanning unit
CCD
Confocal slit
Long pass edge-filter
Beam splitter
Laser scanning mirror
Annotation by certified pathologists
Automated adjustment of microscopic fields
Matrix-based annotation and SERS signal information
Processing pixel-based SERS spectra
IMAGEREVEAL MS software
Tiling multiple stripes of laser scanning SERS images
x y Tag 200 202 204 1750
Wavenumber (cm-1)
1 1 stroma -50 -250 177 315
2 1 stroma -40 -148 66 457
3 1 DCIS -107 -74 220 504
Automated judgement of DCIS or IBC by machine learning of finger-printing SERS spectra (e.g. XGBoost)

Figure S3 SERS signal intensities from whole annotated regions

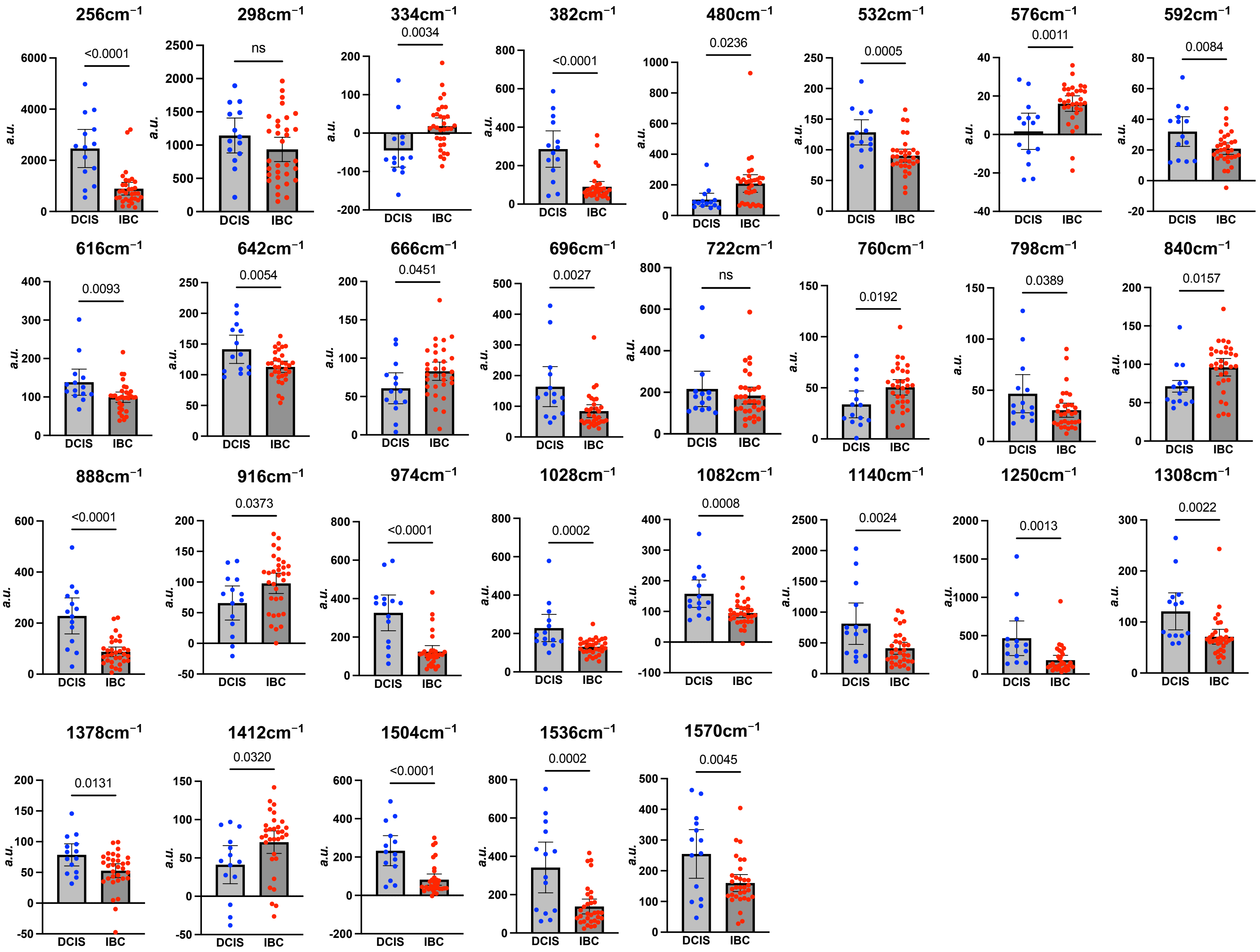

Figure S4 SERS signal intensities from Cancer cell nest

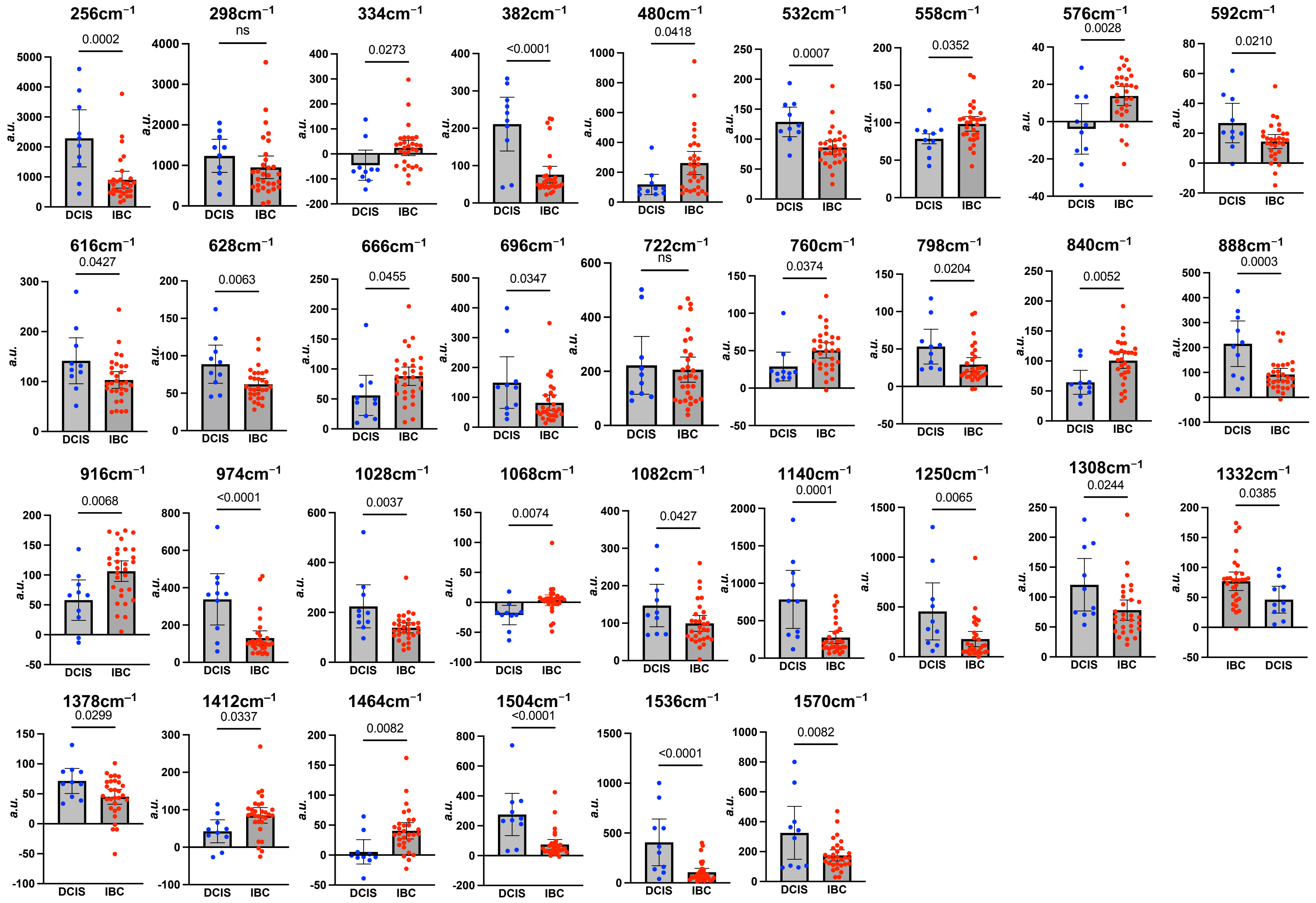

Figure S5 SERS signal intensities from stromal region

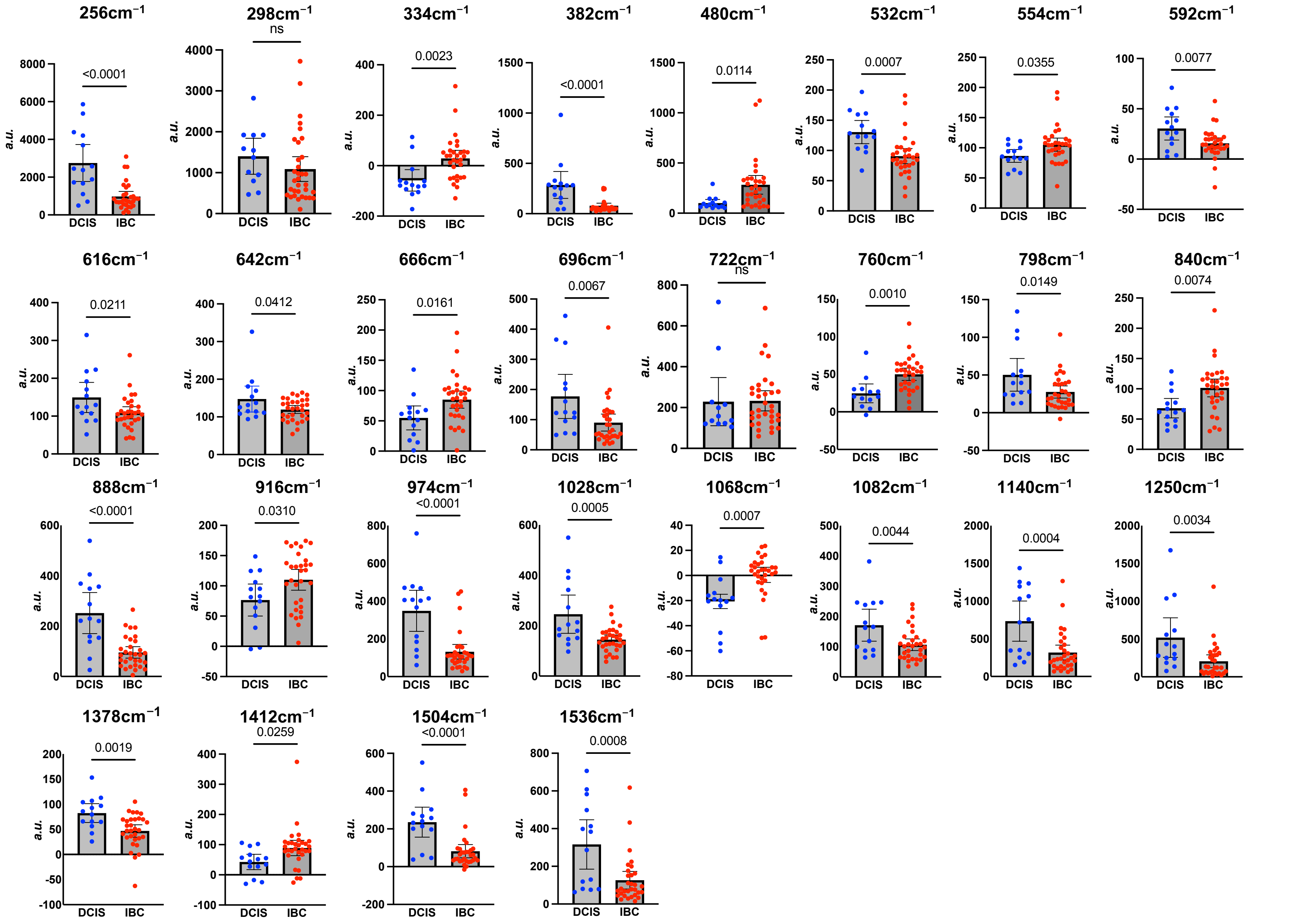

Figure S6 Averaged SERS spectra from adipose tissues

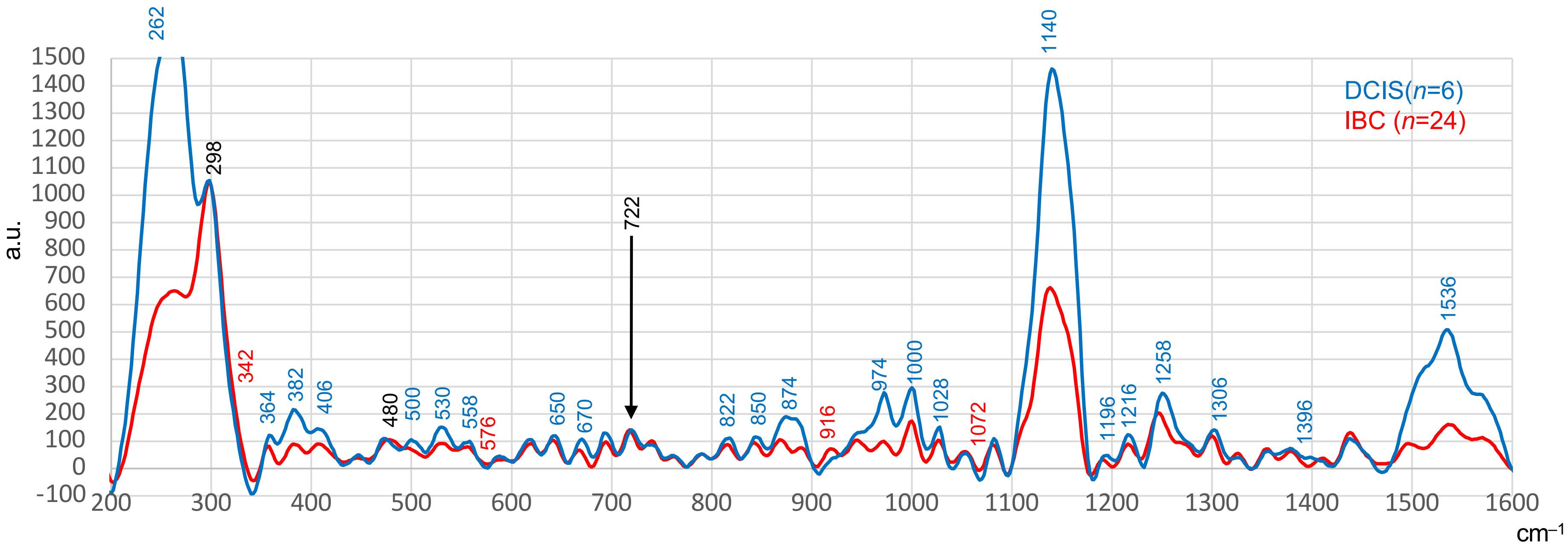

Table S1. Lists of Raman shifts occurring dominantly in frozen section of invasive tumors (I-dominant) and those in DCIS (D-dominant) in the human breast cancer tissue. These raw data collected from the whole annotated tissue region. invasive breast cancer patients (n=32) and DCIS patients (n=14) were statistically analyzed. The shifts with statistical significance (p<0.05) were chosen by unpaired Student's t-test. I mean and D mean indicate mean intensities of the individual Raman shifts at specific wave numbers. The value of dF indicate degree of freedom for unpaired Student's t-test. Resultantly, 8 and 19 different peaks were identified as I-dominant and D-dominant individual shifts, respectively.

cm^{-1}	I mean	D mean	dominant	dF	t-value
256	890.8	2464	D-dominant	44	5.399
298	936.1	1144	not significant	44	1.322
334	18.18	–45.32	I-dominant	44	3.093
382	90.09	286.2	D-dominant	44	5.596
480	208.7	103.7	I-dominant	44	2.346
532	90.55	128.6	D-dominant	44	3.780
576	16.05	1.647	I-dominant	44	3.481
592	20.90	32.05	D-dominant	44	2.761
616	99.38	138.5	D-dominant	44	2.720
642	112.9	141.5	D-dominant	44	2.927
666	83.00	60.75	I-dominant	44	2.062
696	84.44	164.1	D-dominant	44	3.181
722	184.1	116.4	not significant	44	0.811
760	50.55	33.81	I-dominant	44	2.432
798	30.53	46.70	D-dominant	44	2.129
840	96.14	71.35	I-dominant	44	2.514
888	87.56	228.3	D-dominant	44	5.515
916	97.87	65.75	I-dominant	44	2.148
974	124.5	326.1	D-dominant	44	5.536
1028	131.0	228.6	D-dominant	44	4.042
1082	94.61	157.9	D-dominant	44	3.614
1140	413.0	812.9	D-dominant	44	3.216
1250	180.9	466.7	D-dominant	44	3.446
1308	71.07	120.9	D-dominant	44	3.251
1378	52.76	78.60	D-dominant	44	2.587
1412	70.62	41.23	I-dominant	44	2.214
1504	82.34	233.3	D-dominant	44	4.716
1536	138.3	341.9	D-dominant	44	4.128
1570	160.0	254.6	D-dominant	44	2.992

Table S2. Lists of Raman shifts occurring dominantly cancer cell region of invasive tumors (I-dominant) and those in DCIS (D-dominant) in the human breast cancer tissue. These raw data collected from the cancer cells of invasive breast cancer patients (n=30) and DCIS patients (n=10) were statistically analyzed. The shifts with statistical significance (p<0.05) were chosen by unpaired Student's t-test. I mean and D mean indicate mean intensities of the individual Raman shifts at specific wave numbers. The value of dF indicate degree of freedom for unpaired Student's t-test. Resultantly, 12 and 19 different peaks were identified as I-dominant and D-dominant individual shifts, respectively.

cm^{-1}	I mean	D mean	dominant	dF	t-value
256	907.3	2288	D-dominant	38	4.095
298	953.0	1236	not significant	38	1.099
334	24.10	–45.23	I-dominant	38	2.296
382	75.81	211.1	D-dominant	38	5.180
480	262.0	118.4	I-dominant	38	2.107
532	86.18	128.8	D-dominant	38	3.676
558	98.45	78.56	I-dominant	38	2.184
576	13.74	–3.90	I-dominant	38	3.191
592	14.42	26.86	D-dominant	38	2.407
616	103.2	141.6	D-dominant	38	2.098
628	61.84	88.71	D-dominant	38	2.892
666	87.92	55.88	I-dominant	38	2.068
696	82.02	149.5	D-dominant	38	2.190
722	206.1	222.1	not significant	38	0.335
760	50.43	28.92	I-dominant	38	2.157
798	29.20	53.19	D-dominant	38	2.420
840	100.8	64.44	I-dominant	38	2.964
888	91.82	215.1	D-dominant	38	4.012
916	106.4	57.94	I-dominant	38	2.863
974	130.4	337.8	D-dominant	38	4.378
1028	138.8	224.4	D-dominant	38	3.094
1068	3.322	–21.23	I-dominant	38	2.831
1082	99.37	147.3	D-dominant	38	2.096
1140	276.7	784.4	D-dominant	38	4.289
1250	178.5	455.5	D-dominant	38	2.876
1308	78.13	120.6	D-dominant	38	2.343
1332	76.92	46.30	I-dominant	38	2.143
1378	45.00	71.49	D-dominant	38	2.256
1412	84.93	42.37	I-dominant	38	2.203
1464	40.58	5.385	I-dominant	38	2.792
1504	74.47	275.5	D-dominant	38	4.419
1536	106.9	406.4	D-dominant	38	4.466
1570	174.5	325.8	D-dominant	38	2.789

Table S3 Lists of Raman shifts occurring dominantly stroma region of invasive tumors (I-dominant) and those in DCIS (D-dominant) in the human breast cancer tissue. These raw data collected from the cancer stroma of invasive breast cancer patients (*n*=31) and DCIS patients (*n*=14) were statistically analyzed. The shifts with statistical significance ($p<0.05$) were chosen by unpaired Student's *t*-test. I mean and D mean indicate mean intensities of the individual Raman shifts at specific wave numbers. The value of dF indicate degree of freedom for unpaired Student's *t*-test. Resultantly, 9 and 17 different peaks were identified as I-dominant and D-dominant individual shifts, respectively

cm^{-1}	I mean	D mean	dominant	dF	*t*-value
256	978.5	2750	D-dominant	43	4.970
298	1088	1402	not significant	43	1.139
334	28.87	–58.26	I-dominant	43	3.238
382	79.91	285.8	D-dominant	43	4.575
480	282.9	100.5	I-dominant	43	2.643
532	90.75	130.6	D-dominant	43	3.657
554	105.3	86.54	I-dominant	43	2.170
592	15.63	30.42	D-dominant	43	2.796
616	108.9	149.5	D-dominant	43	2.395
642	119.6	147.3	D-dominant	43	2.104
666	85.13	54.90	I-dominant	43	2.506
696	90.38	177.2	D-dominant	43	2.848
722	233	229.1	not significant	43	0.0765
760	49.94	24.55	I-dominant	43	3.534
798	27.51	50.19	D-dominant	43	2.536
840	101.5	68.09	I-dominant	43	2.814
888	95.40	251.7	D-dominant	43	5.211
916	110.0	76.61	I-dominant	43	2.230
974	131.7	348.3	D-dominant	43	4.971
1028	144.8	245.7	D-dominant	43	3.755
1068	0.4685	–20.68	I-dominant	43	3.630
1082	106.4	171.0	D-dominant	43	3.005
1140	317.5	734.4	D-dominant	43	3.816
1250	205.5	519.5	D-dominant	43	3.099
1378	46.81	82.31	D-dominant	43	3.310
1412	88.95	42.62	I-dominant	43	2.308
1504	81.99	235.8	D-dominant	43	4.362
1536	126.4	316.1	D-dominant	43	3.587

Table S4. Baseline data (whole annotated region).

Variable		*n*	Overall, *n*=46[1]	DCIS, *n*=14[1]	IBC, *n*=31[1]	*p*-value[2]
382+/–10 cm⁻¹		46	127(129)	258(140	70(71)	<0.001
480+/–10 cm⁻¹		46	156(123)	98(63)	182(134)	0.006
974+/–10 cm⁻¹		46	132(90)	216(101)	95(53)	<0.001
1140+/–10 cm⁻¹		46	502(398)	762(539)	388(253)	0.025
1250+/–10 cm⁻¹		46	229(398)	389(322)	159(142)	0.021
722+/–10 cm⁻¹		46	161(94)	176(113)	155(86)	0.524
Age		46	58(15)	52(13)	61(15)	0.072
ER		46	34(74%)	11(79%)	23(72%)	0.729
PgR		46	32(70%)	8(57%)	24(75%)	0.301
HER2	negative		21(46%)	2(14%)	19(59%)	<0.001
	positive		8(17%)	1(7.1%)	7(22%)	
	Not determined		17(37%)	11(79%)	6(19%)	
Type	Luminal		31(67%)	10(71%)	21(66%)	0.382
	Triple positive		3(6.5%)	1(7.1%)	2(6.2%)	
	HER2		5(11%)	0(0%)	5(16%)	
	Triple negative		4(8.7%)	1(7.1%)	3(9.4%)	
	Non-luminal		3(6.5%)	2(14%)	1(3.1%)	
Luminal	Non-luminal		9(20%)	3(21%)	6(19%)	>0.999
	Luminal		37(80)	11(79%)	26(81%)	
T	T0		14(30%)	14(100%)	0(0%)	<0.001
	T1		21(46%)	0(0%)	21(66%)	
	T2		9(20%)	0(0%)	9(28%)	
	T3		1(2.2%)	0(0%)	1(3.1%)	
	T4		1(2.2%)	0(0%)	1(3.1%)	
N	N0		41(89%)	14(100%)	27(84%)	0.303
	N1		5(11%)	0(0%)	5(16%)	
M	M0	46	46(100%)	14(100%)	32(100%)	
Stage	Stage0		14(30%)	14(100%)	0(0%)	<0.001
	Stage1		18(39%)	0(0%)	18(56%)	
	Stage2a		11(24%)	0(0%)	11(34%)	
	Stage2b		2(4.3%)	0(0%)	2(6.2%)	
	Stage3b		1(2.52%)	0(0%)	1(3.1%)	
Triple negative	Others		42(91%)	13(93%)	29(91%)	>0.999
	Triple negative		4(8.7%)	1(7.1%)	3(9.4%)	

* [1]Mean (SD); *n* (%), [2]Welch Two Sample *t*-test; Fisher's exact test, DCIS (Non-invasive ductal carcinoma *in situ*), IBC (invasive breast cancer)

Table S5. Baseline data (cancer cell nest region).

Variable		n	Overall, n=40[1]	DCIS, n=10[1]	IBC, n=30[1]	p-value[2]
382+/–10 cm^{-1}		40	89(87)	186(95)	57(56)	0.002
480+/–10 cm^{-1}		40	198(163)	109(80)	227(173)	0.006
974+/–10 cm^{-1}		40	124(97)	218(123)	93(61)	0.011
1140+/–10 cm^{-1}		40	381(363)	735(501)	264(203)	0.016
1250+/–10 cm^{-1}		40	208(232)	372(329)	153(163)	0.069
722+/–10 cm^{-1}		40	176(99)	185(111)	173(96)	0.776
Age		40	59(15)	54(14)	60(15)	0.280
ER		40	30(75%)	8(80%)	22(73%)	>0.999
PgR		40	28(70%)	5(50%)	23(77%)	0.133
HER2	negative		20(50%)	2(20%)	18(60%)	0.009
	positive		8(20%)	1(10%)	7(23%)	
	Not determined		12(30%)	7(70%)	5(17%)	
Type	Luminal		28(70%)	8(80%)	20(67%)	0.480
	Triple positive		2(5.0%)	0(0%)	2(6.7%)	
	HER2		5(12%)	0(0%)	5(17%)	
	Triple negative		3(7.5%)	1(10%)	2(6.7%)	
	Non-luminal		2(5.0%)	1(10%)	1(3.3%)	
Luminal	Non-luminal		7(18%)	2(20%)	5(17%)	>0.999
	Luminal		33(82)	8(80%)	25(83%)	
T	T0		10(25%)	10(100%)	0(0%)	<0.001
	T1		19(48%)	0(0%)	19(63%)	
	T2		9(22%)	0(0%)	9(30%)	
	T3		1(2.5%)	0(0%)	1(3.3%)	
	T4		1(2.5%)	0(0%)	1(3.3%)	
N	N0		35(88%)	10(100%)	25(83%)	0.306
	N1		5(12%)	0(0%)	5(17%)	
M	M0	40	40(100%)	10(100%)	30(100%)	
Stage	Stage0		10(25%)	10(100%)	0(0%)	<0.001
	Stage1		16(40%)	0(0%)	16(53%)	
	Stage2a		11(28%)	0(0%)	11(37%)	
	Stage2b		2(5.0%)	0(0%)	2(6.7%)	
	Stage3b		1(2.5%)	0(0%)	1(9.3%)	
Triple negative	Others	40	37(92%)	9(90%)	28(93%)	>0.999
	Triple negative	40	3(7.5%)	1(10%)	2(6.7%)	

* [1]Mean (SD); n (%), [2]Welch Two Sample t-test; Fisher's exact test, DCIS (Non-invasive ductal carcinoma *in situ*), IBC (invasive breast cancer)

Table S6. Baseline data (stroma region).

Variable		*n*	Overall, *n*=45[1]	DCIS, *n*=14[1]	IBC, *n*=31[1]	*p*-value[2]
382+/–10 cm^{-1}		45	120(159)	255(218)	59(64)	0.005
480+/–10 cm^{-1}		45	198(192)	95(52)	244(214)	<0.001
974+/–10 cm^{-1}		45	136(102)	225(114)	95(64)	<0.001
1140+/–10 cm^{-1}		45	422(361)	691(425)	301(253)	0.005
1250+/–10 cm^{-1}		45	258(281)	434(371)	178(187)	0.026
722+/–10 cm^{-1}		45	193(117)	192(131)	193(112)	0.998
Age		45	58(15)	52(13)	61(15)	0.058
ER		45	33(73%)	11(79%)	22(71%)	0.725
PgR		45	31(69%)	8(57%)	23(74%)	0.307
HER2	negative		21(47%)	2(14%)	19(61%)	<0.001
	positive		8(18%)	1(7.1%)	7(23%)	
	Not determined		16(36%)	11(79%)	5(16%)	
Type	Luminal		29(64%)	9(64%)	20(65%)	0.297
	Triple positive		4(8.9%)	1(7.1%)	3(9.7%)	
	HER2		4(8.9%)	0(0%)	4(13%)	
	Triple negative		4(8.9%)	1(7.1%)	3(9.7%)	
	Non-luminal		4(8.9%)	3(21%)	1(3.2%)	
Luminal	Non-luminal		10(22%)	4(29%)	6(19%)	0.700
	Luminal		35(78%)	10(71%)	25(81%)	
T	T0		14(31%)	14(100%)	0(0%)	<0.001
	T1		20(44%)	0(0%)	20(65%)	
	T2		9(20%)	0(0%)	9(29%)	
	T3		1(2.2%)	0(0%)	1(3.2%)	
	T4		1(2.2%)	0(0%)	1(3.2%)	
N	N0		40(89%)	14(100%)	26(84%)	0.305
	N1		5(11%)	0(0%)	5(16%)	
M	M0	45	45(100%)	14(100%)	31(100%)	
Stage	Stage0		14(31%)1	14(100%)	0(0%)	<0.001
	Stage1		7(38%)	0(0%)	17(55%)	
	Stage2a		11(24%)	0(0%)	11(35%)	
	Stage2b		2(4.4%)	0(0%)	2(6.5%)	
	Stage3b		1(2.2%)	0(0%)	1(3.2%)	
Triple negative	Others	41	41(91%)	13(93%)	28(90%)	>0.999
	Triple negative	4	4(8.9%)	1(7.1%)	3(9.7%)	

* [1]Mean (SD); *n* (%), [2]Welch Two Sample *t*-test; Fisher's exact test, DCIS (Non-invasive ductal carcinoma *in situ*), IBC (invasive breast cancer)

Table S7. Logistic regression models for invasive breast cancer (univariable analyses).

**Characteristic**		*n*	**EVENT *n***	**OR[1]**	**95% CI[2]**	***p*-value[2]**
ER(+)	Whole annotated tissue region	46	32	0.70	0.13, 2.90	N.S.
	Cancer cell nest only	40	30	0.69	0.09, 3.52	N.S.
	Stroma region only	45	31	0.67	0.13, 2.78	N.S.
PgR(+)	Whole annotated tissue region	46	32	2.25	0.59, 8.64	N.S.
	Cancer cell nest only	40	30	3.29	0.73, 15.4	N.S.
	Stroma region only	45	31	2.16	0.56, 8.30	N.S.
HER2(+)	Whole annotated tissue region	46	32	0.74	0.06, 17.3	N.S.
	Cancer cell nest only	40	30	0.78	0.06, 18.3	N.S.
	Stroma region only	45	31	0.74	0.06, 17.3	N.S.
Luminal	Whole annotated tissue region	46	32	1.18	0.22, 5.39	N.S.
	Cancer cell nest only	40	30	1.25	0.16, 7.17	N.S.
	Stroma region only	45	31	1.67	0.36, 7.18	N.S.
Triple negative	Whole annotated tissue region	46	32	1.34	0.15, 28.6	N.S.
	Cancer cell nest only	40	30	0.64	0.05, 14.8	N.S.
	Stroma region only	45	31	1.39	0.16, 29.7	N.S.

* [1]OR = Odds Ratio, [2]CI = Confidence Interval

Table S8. Lists of Raman shifts occurring dominantly in adipose tissue region of invasive tumors (I-dominant) and those in DCIS (D-dominant) in the human breast cancer tissue. These raw data collected from the adipose tissue of invasive breast cancer patients (n=24) and DCIS patients (n=6) were statistically analyzed. The shifts with statistical significance (p<0.05) were chosen by unpaired Student's t-test. I mean and D mean indicate mean intensities of the individual Raman shifts at specific wave numbers. The value of dF indicate degree of freedom for unpaired Student's t-test. Resultantly, 4 and 22 different peaks were identified as I-dominant and D-dominant individual shifts, respectively.

cm^{-1}	I mean	D mean	dominant	dF	t-value
262	349.3	1688	D-dominant	28	2.988
342	–44.89	–92.04	I-dominant	28	3.499
364	30.54	95.74	D-dominant	28	2.957
382	89.75	215.7	D-dominant	28	3.708
406	89.70	145.5	D-dominant	28	4.563
500	69.89	105.2	D-dominant	28	3.010
530	91.23	151.2	D-dominant	28	3.572
558	77.26	99.92	D-dominant	28	2.670
576	16.13	2.456	I-dominant	28	2.671
650	54.06	73.72	D-dominant	28	2.932
670	62.29	107.3	D-dominant	28	3.252
822	59.84	90.53	D-dominant	28	4.057
850	62.40	96.19	D-dominant	28	2.578
874	88.72	188.6	D-dominant	28	4.354
916	65.13	19.15	I-dominant	28	2.403
974	94.30	271.3	D-dominant	28	5.777
1000	174.6	295.5	D-dominant	28	2.318
1028	101.7	151.2	D-dominant	28	3.460
1072	14.00	–17.39	I-dominant	28	3.148
1140	655.7	1461	D-dominant	28	3.293
1196	17.89	47.51	D-dominant	28	2.799
1216	88.73	123.9	D-dominant	28	2.097
1258	123.4	226.4	D-dominant	28	2.182
1306	78.63	123.6	D-dominant	28	3.562
1390	20.88	43.48	D-dominant	28	3.540
1536	160.8	507.0	D-dominant	28	3.827

Table S9 RESULT in model training.

**XGB**		**Diagnosed class labels**	
		**DCIS**	**IBC**
**Predicted**	**DCIS**	285	7
**class labels**	**IBC**	13	2812

The prediction of the XGBoost model on test-fold dataset in three-fold cross validation. The confusion matrix shows only seven invasive patients-derived meshes out of 2,819 were mislabeled.
